# PET molecular imaging for pathophysiological visualization in Alzheimer’s disease

**DOI:** 10.1007/s00259-022-05999-z

**Published:** 2022-11-14

**Authors:** Jing Wang, Chentao Jin, Jinyun Zhou, Rui Zhou, Mei Tian, Hyeon Jeong Lee, Hong Zhang

**Affiliations:** 1 Department of Nuclear Medicine and PET Center, The Second Affiliated Hospital of Zhejiang University School of Medicine, 88 Jiefang Road, Hangzhou, 310009 Zhejiang China; 2 Institute of Nuclear Medicine and Molecular Imaging of Zhejiang University, Hangzhou, 310009 Zhejiang China; 3 Key Laboratory of Medical Molecular Imaging of Zhejiang Province, Hangzhou, 310009 Zhejiang China; 4 College of Biomedical Engineering and Instrument Science, Zhejiang University, Hangzhou, 310014 Zhejiang China; 5 Key Laboratory for Biomedical Engineering of Ministry of Education, Zhejiang University, Hangzhou, 310014 Zhejiang China

**Keywords:** Alzheimer’s disease, Positron emission tomography (PET), Molecular imaging

## Abstract

Alzheimer’s disease (AD) is the most common dementia worldwide. The exact etiology of AD is unclear as yet, and no effective treatments are currently available, making AD a tremendous burden posed on the whole society. As AD is a multifaceted and heterogeneous disease, and most biomarkers are dynamic in the course of AD, a range of biomarkers should be established to evaluate the severity and prognosis. Positron emission tomography (PET) offers a great opportunity to visualize AD from diverse perspectives by using radiolabeled agents involved in various pathophysiological processes; PET imaging technique helps to explore the pathomechanisms of AD comprehensively and find out the most appropriate biomarker in each AD phase, leading to a better evaluation of the disease. In this review, we discuss the application of PET in the course of AD and summarized radiolabeled compounds with favorable imaging characteristics.

## Introduction

Alzheimer’s disease (AD) is a progressive neurodegenerative disorder characterized by gradual memory loss and cognitive decline [1]. It is the most common dementia worldwide, accounting for 50–60% of all cases (World Alzheimer Report 2018, www.alz.co.uk). Though some population-based studies suggest a decreased incidence of AD in high-income countries [2–5], the prevalence is increasing constantly. Approximately 30 million people are now estimated to live with AD, and the number is projected to triple by 2050 [6]. Despite great efforts have been made to explore the underlying pathological mechanisms and disease-modifying treatments, there is no effective therapy for AD [7], resulting in a substantial burden posed to the whole society.

Diagnosis of AD now depends on clinical phenotypes and some biomarkers suggesting the presence of Alzheimer’s pathology [8, 9]. However, in the first panel of diagnostic criteria for AD launched in 1984, due to a lack of reliable means to detect neuropathological changes, only clinical symptoms were focused, and definite diagnosis was hard to make until autopsy [10]. Over the last few decades, with the advancement in structural and functional imaging techniques, the pathological processes of AD are being revealed gradually. While structure imaging biomarkers combining with clinical phenotypes allow a sensitive and specific diagnosis of AD in mid or late stages [11, 12], researchers are gradually moving forward to explore more subtle changes in earlier phases.

Functional imaging, especially molecular imaging, compared with traditional means, is considered as a more sensitive neuroimaging modality, offering delicate pathophysiological information long before structural changes were presented [13], which may promote traditional pathology to “transpathology” [14]. Positron emission tomography (PET) is a representative technique of molecular imaging, permitting in vivo quantification of radioisotopes in nanomolar to picomolar range [15, 16]. And by using diverse radiotracers, analogs, or substrates of target processes, PET can be used to assess protein binding, receptor availability, transporter systems, signal transduction, and gene expression [15]. Thus, PET presents great potential in AD management, no matter for early diagnosis, disease progression monitoring, or therapeutic effect evaluation [17–19].

The improvements of imaging techniques have led imaging biomarkers to be incorporated into diagnostic criteria of AD. In addition to some biochemical markers in cerebrospinal fluid (CSF), the National Institute on Aging and the Alzheimer’s Association (NIA-AA) also introduced a panel of imaging biomarkers into the new guidelines in 2011. Atrophy of mesial temporal lobe on MRI, amyloid-beta deposition on amyloid PET, and hypometabolism on ^18^F-fluorodeoxyglucose (FDG) PET were used to assess the likelihood that clinical symptoms is due to AD and determine the stage of preclinical AD [8, 13, 20]. After that, the International Working Group (IWG) also proposed an adjusted version of diagnostic criteria for AD, in which the level of Aβ plaques quantified by PET was considered as pathological biomarker of AD, while hypometabolism in ^18^F-FDG PET and brain atrophy in MRI were regarded as biomarkers of disease progression [9].

These two new guidelines both emphasized the crucial role of imaging biomarkers in advancing preclinical AD research, since this may finally aid the development of disease-modifying interventions. As numerous studies show, clinical signs of most AD patients emerge after an extensive preclinical period, which can reach up to 15–20 years [21, 22]. Over such a prolonged preclinical period, using some non-invasive measures to monitor the occurrence and progression of AD is of critical value. And considering the most fitting indicator may vary in different preclinical phases [13, 23], a range of various biomarkers should be developed and utilized in different stages. In this review, we describe the application of PET as well as its importance in different AD stages and list main categories of PET radiotracers available now (Tables 1 and 2).

**Table 1:** PET molecular imaging to study major AD pathophysiology

Pathophysiology	Targets	Radiotracers	Advantages	Limitations	References
Aβ	Fibrillar Aβ	^11^C-PIB	High affinity for Aβ-fibrils (Ki = 20.2 nM); high sensitivity and specificity; better accuracy than CSF biomarkers for AD diagnosis and progression predicting	Short half-life (20 min)	[24–26]
		^18^F-florbetapir, ^18^F-flutemetamol, ^18^F-florbetaben	Longer half-life (110 min); high sensitivity and specificity	Appreciable white matter retention may hamper the visual assessment	[19, 27–30]
		^18^F-AZD4694	High affinity for Aβ-fibrils (*K* (*d*) = 2.3 + / − 0.3 nM); high sensitivity and specificity; favorable kinetic profile and relatively low test–retest variability	-	[31–33]
	Prefibrillar Aβ	^125^I-mAb158	High affinity for soluble protofibrils	-	[34]
		^124^I-di-scFv-3D6-8D3	Ability to cross the blood–brain barrier and bind to both fibrillary Aβ and soluble aggregates	-	[35, 36]
Tau	Tau	^18^F-AV-1451	High selectivity and specificity; high affinity to PHF-tau (Kd = 14.6 nM); favorable tracer kinetics; good reproducibility; high accuracy for AD diagnosis; better than ^11^C-PIB to correlate to the severity of cognitive impairment	The kinetics varies in different disease stages; the uptake curve does not plateau; off-target binding	[37–43]
		^18^F-AV-680	High selectivity and affinity to PHF-tau; little white matter retention and minimal off-target binding	-	[44]
		^18^F-THK-5105, ^18^F-THK-5117	Improved tau affinity and selectivity	Nonspecific binding	[45, 46]
		^18^F-THK-5351	Favorable pharmacokinetics; higher affinity for tau protein fibrils; lower retention in white matter	Less sensitive and specific to tau and higher off-target binding than ^18^F-AV-1451; affected by the availability of MAO-B	[47–49]
		^11^C-PBB3	Good binding affinity; high selectivity; ability to detect a broad range of tau aggregates	Noticeable retention in venous sinuses; instability	[50–52]
Glucose metabolism		^18^F-FDG	Differentiate AD from healthy subjects and other dementias; predict the conversion from MCI to AD	Susceptible to many external factors; offers less information about disease etiology	[53–58]

Abbreviations: *Aβ* amyloid-beta, *AD* Alzheimer’s disease, *CSF* cerebrospinal fluid, *MAO-B* monoamine oxidase B, *PET* positron emission tomography, *PHF* paired helical filament

**Table 2:** PET molecular imaging for minor AD pathophysiology

Hallmarks	Targets	Radiotracers	References
Neuroinflammation	18-kDa translocator protein	^11^C-PK11195, ^11^C-DAA1106, and ^18^F-FEDAA1106	[59–61]
	MAO-B	^11^C-DED	[62]
	Phospholipase activity	^11^C-AA	[63]
Metal ions in amyloid plaques	Metallobiology	^18^F-CABS13, ^11^C-L2-b, and ^18^F-FL2-b	[64, 65]
GSK3		^11^C-A1070722, ^11^C-labelling isonicotinamides, and ^18^F-maleimide-based ligands	[66–69]
Glutamatergic systems	mGluR2	^11^C-JNJ42491293	[70]
	mGluR1	^18^F-FITM, ^11^C-ITMM, ^11^C-ITDM, and ^18^F-FIMX	[70]
	mGluR5	^18^F-FPEB, ^18^F-SP203, and ^11^C-ABP688	[71]
Endogenous cannabinoid system	CB1R	^18^F-MK-9470, ^18^F-FMPEP-d2, 18F-FPATPP, and 11C-SD5024	[72–75]
	CB2R	^18^F-labelling 1,3,5-triazine derivatives or ^11^C-labelling 4-oxoquinoline derivatives	[76]
	FAAH	^11^C-CURB, ^11^C-MK-3168, and 11C- FAAH-1906	[77–79]
Cholinergic system	AChE activity	^11^C-PMP and ^11^C-MP4A	[80, 81]
	mAChR	^11^C-MK-6884	[82]
	nAChR	18F-ASEM	[83]
	VAChT	^11^C-TZ659, ^18^F-VAT, and radiolabelling benzovesamicol VAChT analogs	[84–86]
Monoaminergic system	5-HT_1A_ receptor	^11^C-WAY100635 and ^18^F-MPPF	[87, 88]
	NE	^11^C-MRB	[89]

Abbreviations: *5-HT* serotonin, *AChE* acetylcholinesterase, *CB1R* type 1 cannabinoid receptor, *CB2R* type 2 cannabinoid receptor, *FAAH* fatty acid amide hydrolase, *GSK3* glycogen synthase kinase 3, *mAChR* muscarinic acetylcholine receptors, *MAO-B* monoamine oxidase B, *mGluRs* metabotropic glutamate receptors, *nAChR* nicotinic acetylcholine receptors, *NE* norepinephrine, *VAChT* vesicular acetylcholine transporter

## Amyloid-beta imaging

Amyloid-beta (Aβ) is a kind of peptide critically involved in AD pathophysiology, as components of the amyloid oligomers, fibrils, and plaques [90]. Aβ derives from amyloid precursor protein (APP), an integral membrane protein regulating synapse formation [91] and neural plasticity [92], after being sequentially cleaved by β-site APP-cleaving enzyme 1 (BACE1) and γ-secretase complex [93]. And the elimination of the Aβ depends on various clearance systems in brain via degradation, blood–brain barrier transport, interstitial fluid drainage, or CSF absorption [94]. Once the balance of Aβ metabolism is damaged, toxic conformations of Aβ would emerge and trigger pathological processes [95, 96].

There is substantial evidence to support that the accumulation of abnormally folded Aβ is closely related to progressive brain atrophy and cognitive impairment [97, 98]. And many neurological effects presented in Aβ-harbored individuals are similar to those in AD patients [99]. As now dominantly hypothesized, amyloidosis is the initial event of AD, followed by a cascade of pathophysiological processes including the formation of neurofibrillary tangles (NFTs), neuroinflammation, synaptic dysfunction, and eventually neuronal death [100–103]. In subjects with Aβ pathology cognitive intact, those with high Aβ burden often present slightly inferior neuropsychological performance and a higher likelihood of disease progression [104–107]. And as suggested by a longitudinal study, approximately 82% of mild cognitive impairment (MCI) patients presenting Aβ-positive would finally progress to AD during the follow-up period, while the majority of Aβ-negative subjects being cognitively stable [108]. Thus, Aβ burden is a reliable indicator of preclinical AD phase and disease progression.

Currently, two major biomarkers are available to characterize Aβ accumulation: Aβ_42_ in CSF and Aβ burden characterized by PET imaging [93]. CSF Aβ_42_ is quite a specific marker of AD, showing a robust inverse correlation with amyloid load in brain [109], and a good accuracy to differentiate AD from age-matched controls [110]. However, as lumbar puncture is a painfully invasive procedure, it may not be the best method for repeated measurements in longitudinal studies. Instead, amyloid PET allows non-invasive evaluation of cerebral Aβ load. Since amyloid PET shows a similar diagnostic accuracy and higher specificity than CSF Aβ_42 _[111], it could be a better choice for AD research. Thanks to the great effort made in the developing of radiotracers to visualize Aβ in vivo, the field of amyloid imaging has been growing fast. We summarized some radioligands of interest here.

### ^***11***^***C-PIB: the first selective Aβ tracer***

^11^C-Pittsburgh compound B (^11^C-PIB) is the first and one of the most successful selective Aβ tracers after a non-specific radiotracer named ^18^F-FDDNP, which greatly drives AD researches forward. As a derivative of Thioflavin T, a dye widely used to visualize amyloid in vitro, ^11^C-PIB could bind to beta-sheet structures, especially fibrillar Aβ in plaques with high affinity (Ki = 20.2 nM) [24]. To some extent, ^11^C-PIB also binds to Aβ oligomers, a neurotoxic form of Aβ gaining increasing attention [112]. Though ^11^C-PIB was found bind to tau [113] and α-synuclein [114] as well, in the concentrations clinically used, neurofibrillary tangles [115] and Lewy bodies [114] do not contribute to the retention of PIB. These characteristics lead to a sensitive and specific quantification of Aβ burden in vivo.

A robust inverse correlation was observed between ^11^C-PIB retention and Aβ_42_ level in CSF [116]. And in comparison with CSF biomarkers, PET techniques greatly improved the accuracy of clinical diagnosis [25]. As postmortem studies suggested, the retention of ^11^C-PIB in PET images is highly correlated to the distribution areas of Aβ at autopsy [117]. Similar to studies conducted by Braak, AD patients mostly show increased uptake in frontal, cingulate, precuneus, striatum, parietal, and lateral temporal cortices. And regions of occipital, sensorimotor, and mesial temporal lobe are usually spared [7]. The striking similarity in the lobes affected and cortices supporting memory function further suggests the importance of amyloid PET in indicating early AD stages [118]. Besides, as amyloid distribution is different in various dementias [119, 120] and AD subtypes [7], the spatial patterns of amyloid deposition measured by PET may help to differentiate AD from other neurodegenerative diseases and determine AD phenotypes.

Additionally, ^11^C-PIB positive is a sensitive predictor of disease conversion from MCI to AD [26]. With the faster converters commonly showing higher PIB uptake in brain [108], PET presents a better accuracy than CSF biomarker to classify stable MCI and progressed MCI [121]. And the specificity of conversion predicting would constantly increase as the follow-up period elongates [122]. Thus, ^11^C-PIB PET has been applied to help researchers select subjects of preclinical AD, of which the group is extensively focused on as the target of Aβ-related therapies. ^11^C-PIB also possesses great potential to assess treatment response, providing much crucial information about the reduction of amyloid burden and its effects on cognitive decline [123].

### ^***18***^***F-florbetapir, ***^***18***^***F-flutemetamol, and ***^***18***^***F-florbetaben: the second generation of Aβ tracers***

Though ^11^C-PIB possesses plenty of virtues, the short half-life of carbon-11 (half-life of 20 min) limits its access only in PET centers with an on-site cyclotron and experienced carbon-11 radiochemistry technicists. To solve this dilemma, multiple fluorine-18 (half-life of 110 min) labeled radiotracers were developed, including ^18^F-florbetapir (a.k.a. Amyvid) [27], ^18^F-flutemetamol (a.k.a. Vizamyl) [28], and ^18^F-florbetaben (a.k.a. Neuraseq) [29]. They were known as the second generation of amyloid imaging radiotracers. And to date, these compounds have been approved by the US Food and Drug Administration (FDA). The longer half-life of fluorine-18 enables these tracers to be centrally produced and regionally distributed like ^18^F-FDG, which may greatly lower the cost and allow AD researches be extensively conducted.

Research using these three tracers have confirmed their high sensitivity and specificity [19]. And mounting evidence has suggested their potential role in amyloidosis early detecting, AD subjects discriminating, and disease progression prediction [7]. In general, results derived from ^11^C-PIB could mostly be replicated. Although different binding characteristics can be observed both in white matter and grey matter, cortical retention of these tracers was highly correlated with that of ^11^C-PIB. The strong link between them may allow PET data be transformed and compared in multicenter studies that use different tracers [124].

These tracers have been extensively adopted by researchers around the world, and few drawbacks were reported. But in a minority of subjects, appreciable white matter retention can be observed, which may hamper the visual assessment of clinicians [30]. And according to a study evaluating the effect of concurrent pathologies on the uptake of florbetapir, while other diseases do not affect the tracer retention, Lewy bodies significantly lower the ratio of standard uptake value (SUV), the underlying mechanisms worth further exploring [125].

### ^***18***^***F-NAV4694: potentially novel Aβ tracer***

**Fig. 1:**
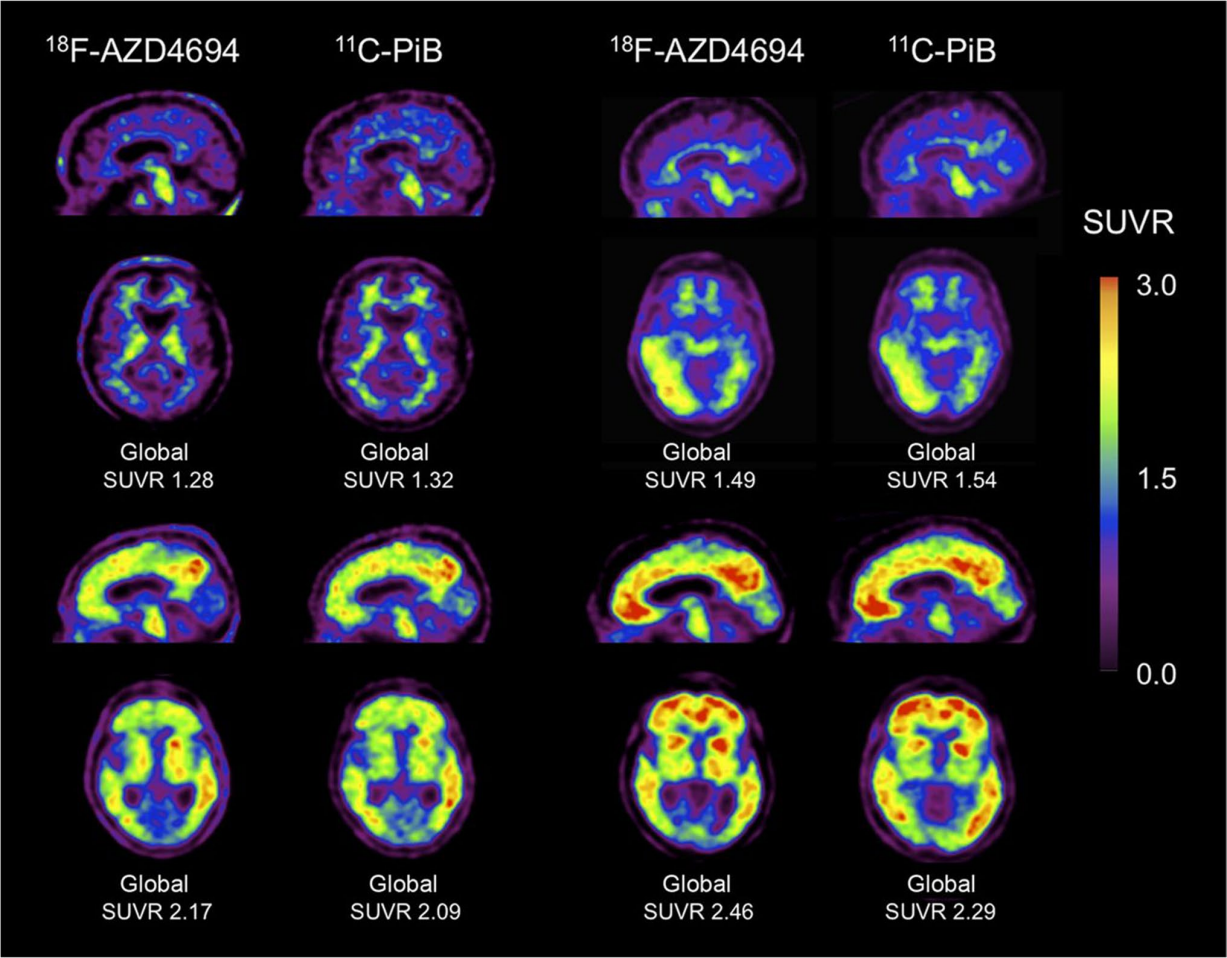
^18^F-AZD4694 and ^11^C-PiB PET imaging in 4 subjects. Four examples of sagittal and transaxial ^18^F-AZD4694 images adjacent to same-slice ^11^C-PiB images in same subject. Top 2 subjects were healthy age-matched controls, and bottom 2 were AD patients. The images showed near-identical appearance and dynamic range of SUVRs of ^18^F-AZD4694 and.^11^C-PiB. (This research was originally published in Journal of nuclear medicine: official publication, Society of Nuclear Medicine. 2013;54:880–6. Reprint permission was obtained)

^18^F-NAV4694, formerly known as ^18^F-AZD4694, is a more novel radiotracer being considered to outperform ligands currently available. In vitro studies of AZD4694 have shown its high affinity for Aβ-fibrils (*K* (*d*) = 2.3 + / − 0.3 nM). And in brain tissues of AD patients, selective binding to Aβ deposits of NAV4694 can be observed in grey matter, while the non-specific signal in white matter is low [31]. Besides, according to results of clinical validation, ^18^F-NAV4694 possesses a favorable kinetic profile and a relatively low test–retest variability [32]. When comparing ^18^F-NAV4694 with ^11^C-PIB, nearly identical imaging characteristics were reported (Fig. 1) [33]. All these results suggest the tremendous potential of ^18^F-NAV4694 to be widely applied both in clinical practice and scientific research.

### *Prefibrillar Aβ imaging*

It is worth noting that most radioligands available now are developed to bind to Aβ in insoluble fibrils. However, increasing evidence has suggested that soluble Aβ aggregates are the neurotoxic form of Aβ causing neural dysfunction. Moreover, diffuse plaque pathology in AD patients usually presents low PIB retention in brain due to the relatively low level of fibrillar Aβ [126]. Soluble Aβ usually better correlated to the severity of AD than amyloid plaques [127]. These results call for agents to visualize soluble oligomers and further investigate their role in AD pathology. In 2003, an antibody-based radioactive ligand, ^125^I-mAb158, was reported to show high affinity for soluble protofibrils [34]. And recently, some derivatives of this radioligand were described in both diagnostic and therapeutic research. ^124^I-RmAb158-scFv8D3 was used to evaluate the distribution of an antibody RmAb158-scFv8D3 in a protofibril-clearing immunotherapy research [36]. And ^124^I-Di-scFv-3D6-8D3 was developed with the ability to cross the blood–brain barrier and bind to both fibrillary Aβ and soluble aggregates [35].

### *Limitations*

Although amyloid PET has contributed much to a better understanding of AD pathophysiology, some problems remain unsolved. Histologically determined Aβ deposition was reported to poorly correlate to regional uptake in some PIB negative patients, calling for further postmortem research to evaluate the binding characteristics [128]. As there are some other mechanisms involved in disease progression [129], the dynamic alternation of amyloid pathology and its correlation with other biomarkers should be further explored. Besides, according to some studies comparing amyloid PET and other measures, PET is a sensitive technique to predict disease progression with limited specificity. So other biomarkers such as ^18^F-FDG metabolism or brain atrophy should be taken into account to improve the prediction specificity [130, 131]. Additionally, different PET centers might apply various protocols to complete Aβ imaging acquisition. Meanwhile, the interpretation of PET images relied on the clinical experience of physicians. Therefore, more standardized approaches involved in image acquisition and interpretation could be helpful to reduce the variation. A guideline or widely accepted protocol could assist physicians to perform a standardized Aβ imaging acquisition and minimize errors produced by operators or PET machines. The combination of visual interpretation and SUVR in Aβ imaging interpretation could establish a semiquantitative analysis system which decreased the variation developed by visual interpretation to some extent [132]. And as studies of prefibrillar Aβ imaging are rather few so far, more research is warranted to develop better prefibrillar Aβ tracers in the future.

## Tau imaging

Tau protein is a multifunctional protein mainly locating in axons of central nervous system, playing a crucial role in stabilizing microtubules [133], which is critical for the neuron integrity and axonal transport [134]. Six tau isoforms are existing in human brain, generated by alternative mRNA splicing of the microtubule-associated protein tau gene (MAPT). And these isoforms can be classified as either 3 (3R) or 4 (4R) repeated binding domains, which may have different functions in various brain areas [135]. Naturally, tau is unfolded and highly soluble, showing little tendency to aggregate. However, the phosphorylation of tau will hamper the affinity of tau for microtubules and make them disassembled, impairing the function of microtubules. Then, the hyperphosphorylated tau aggregate and result in the formation of paired helical filaments (PHFs) and straight filaments. The neurofibrillary tangles (NFTs) formed by PHFs serve as one of the most essential pathological hallmarks of Alzheimer’s disease, derailing neuronal functions and causing cell death [133]. 3R and 4R isoforms are both present in Alzheimer’s disease, while one dominant type is more common in other tauopathies [136].

Though amyloid-beta deposition is considered as the initial event of AD, the lag between the onset of Aβ pathology and the emergence of cognitive decline is usually long. And the correlation between Aβ burden and clinical disease stage gradually weakens after the initial phase of the preclinical stage of AD [13]. Thus, it is necessary to develop another biomarker to evaluate subsequent pathological changes and monitor disease progression. The accumulation of tau could be such an appropriate indicator. Post-mortem studies have shown that the burden of neocortical NFTs is highly associated with cognitive dysfunction [137]. More recent research also further suggest the central role of tau in AD pathophysiology [138]. And the failure of substantial anti-amyloid trials also strengthens the doubt of amyloid hypothesis, calling for a novel perspective to understand AD pathology [139]. It is of great value to explore the tau protein as a diagnostic or therapeutic target.

Tau in CSF, total tau (t-tau) and phosphorylated tau (p-tau), are widely used as core CSF biomarkers, with CSF p-tau highly correlating with the amount of phosphorylated tau in brain and CSF t-tau reflecting the intensity of neuronal degeneration [140]. But tau pathophysiology is also a biomarker of many other neurodegenerative disease, including corticobasal degeneration (CBD), frontotemporal dementia (FTD), progressive supranuclear palsy (PSP), and Pick’s disease. Considering the distribution of tau aggregates differs from each other in these dementias [141], using imaging techniques rather than CSF biochemical biomarkers to visualize the level of tau burden and the spatial distribution seems more anticipated and powerful. Furthermore, it is suggested that the progression of tau deposition in AD follows a stereotypical pattern, with tau deposition limited in the transentorhinal region in early phase, then affecting limbic lobes and finally spreading to neocortical areas [142]. So, tau-targeted PET imaging may better help clinicians staging AD pathology and predicting MCI prognosis as well [143].

However, the developing of tau-binding PET compounds is more challenging than that of Aβ-binding ligands [144]. As tau pathology locates intracellular, binding to tau requires radiotracers to cross both the blood–brain barrier and cell membranes. Moreover, due to the diversities in isoforms and conformations of tau, and a much lower concentration of tau-aggregates than Aβ in brain, ligands used should be much more sensitive and selective. Several series of compounds are available now as potential tau-targeted PET radiotracers, the benzimidazole-pyrimidines derivatives: ^18^F-AV-1451 (a.k.a. ^18^F-T807 and Flortaucipir) and ^18^F-AV-680 (a.k.a. ^18^F-T808); the quinoline derivatives: ^18^F-THK-5105, ^18^F-THK-5117, and ^18^F-THK-5351; and the pyridinyl-butadienyl-benzothiazoles derivative: ^11^C-PBB3. In addition to these tracers, some more novel compounds, such as ^18^F-MK-6240 [145], ^18^F-RO-6958948 [146], also showed excellent properties for tau imaging, but further researches are needed. These agents are increasingly being used, greatly deepening our understanding of AD tau pathophysiology.

### ^***18***^***F-AV-1451 and ***^***18***^***F-AV-680: PHF-tau binding radiotracers***

^18^F-AV-1451 (a.k.a. ^18^F-T807 or ^18^F-flortaucipir) was the first promising PET tracer for visualization and quantification of NFT pathology in AD patients. And to date, it has been the most widely used tau-binding radiotracer [37]. According to results of autoradiography and immunohistochemistry staining in human cortical sections, the selectivity of ^18^F-AV-1451 for tau is 29-fold over Aβ. It also presents high affinity to PHF-tau in the nanomolar range (Kd = 14.6 nM), very low non-specific binding in white matter, and favorable tracer kinetics [37]. Moreover, good reproducibility was reported in a test–retest study, making ^18^F-AV-1451 PET a powerful means in longitudinal researches [38, 39].

**Fig. 2:**
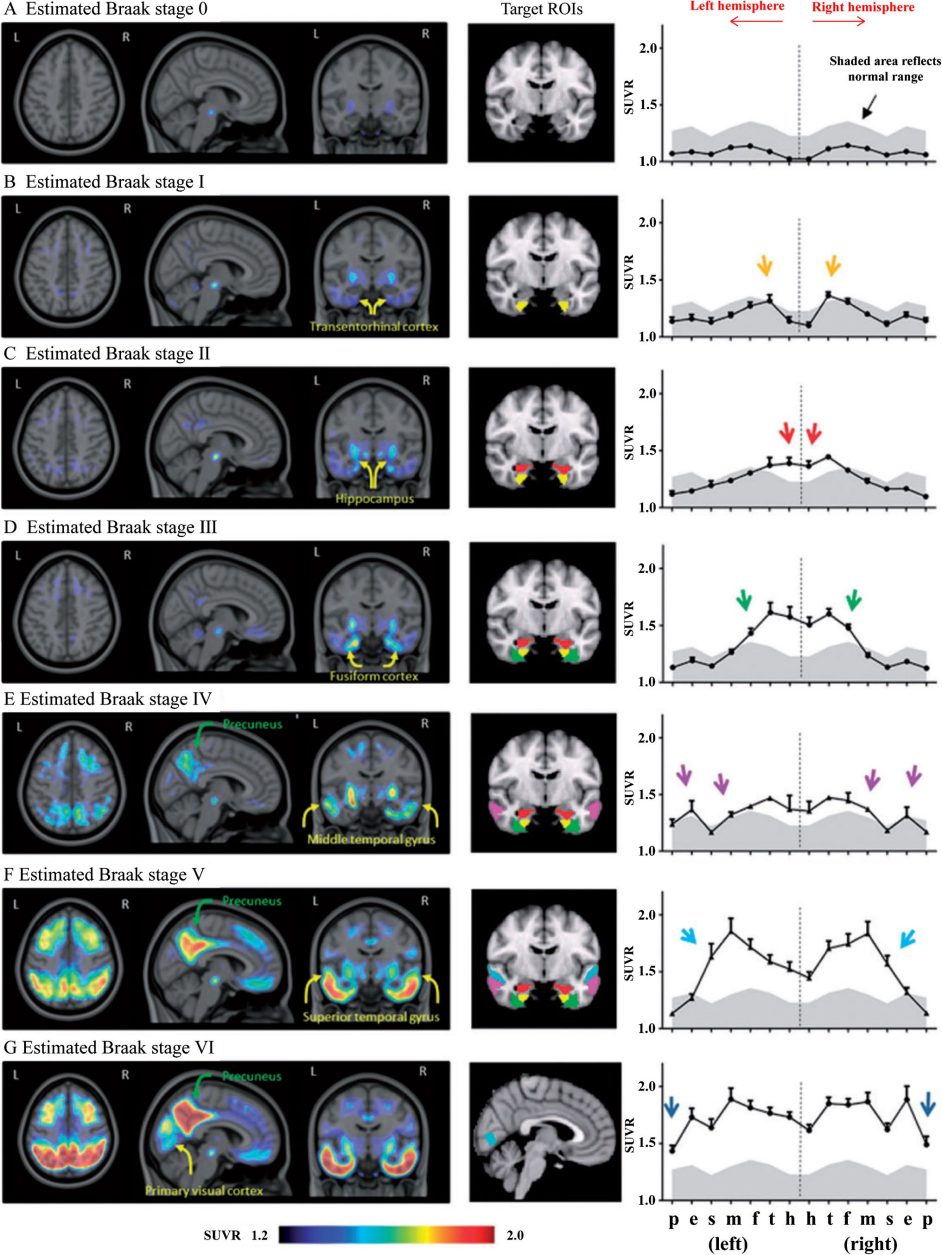
Average SUVR images of.^18^F-AV-1451 PET scans and region of interest profiles by estimated Braak stage. The regions of interest profiles are as showed “butterfly plots,” with the regions of interest from each hemisphere mirrored and ordered mediolaterally, reflecting both the anatomical distribution and order of involvement consistent with disease progressiveness. (This research was originally published in Brain. 2016;139:1539–50. Reprint permission was obtained)

Studies comparing tau PET imaging and CSF biomarkers revealed a significant correlation between tau in CSF and ^18^F-AV-1451 uptake, especially in some specific cortical regions [147]. And in patients with memorial dysfunction, ^18^F-AV-1451 is able to distinguish AD from other non-AD neurodegenerative disorders with high accuracy [40]. Abnormally higher cortical retention can be observed in MCI and AD patients compared with cognitive normal subjects, and the elevated binding of ^18^F-AV-1451 is better correlated to the severity of cognitive impairment than ^11^C-PIB [148]. Besides, it is suggested that the distribution of tracer retention can be classified into patterns as Braak prescribed (Fig. 2) [149], enabling tau PET to monitor progression of tau pathology and evaluate the cause of clinical symptoms. ^18^F-AV-1451 positive results can also be observed in preclinical AD patients, with faster increase of tracer retention being observed in the group of high amyloid burden [150], which may further suggest a close relationship between tau and amyloid-beta, making ^18^F-AV-1451 PET a valuable method to screen and determine subjects in disease-modifying clinical trials. And more recently, international consensus suggested to consider the application of ^18^F-AV-1451 PET imaging for definitive diagnosis, differential diagnosis, and severity determination of AD [151].

However, this tracer has some limitations. As a study has suggested, the kinetics of this tracer in brain varies in different disease stages, and the uptake curve does not plateau during the typical imaging duration; further dynamic researches are warranted to determine the best timing of scanning for accurately diagnosing [41]. Additionally, in some cortical areas with no tau pathology, off-target binding of ^18^F-AV-1451 was reported, especially in basal ganglia, substantia nigra, and choroid plexus [42, 43], so more imaging-pathological studies on postmortem materials should be conducted for a better interpretation of PET imaging.

^18^F-AV-680 is another benzimidazole-pyrimidines derivative showing promise for the tau imaging in AD patients. It also displays high selectivity and affinity to PHF-tau, little white matter retention, and minimal off-target binding. In contrast to ^18^F-AV-1451, ^18^F-AV-680 demonstrates a substantial defluorination in rodents, and a faster kinetics in human brains [44]. But studies are warranted to further verify these findings.

### *THK series: quinoline derivatives for tau PET imaging*

The first ^18^F-labelling quinoline derivatives created for tau imaging were ^18^F-THK523, but its clinical application was hampered due to several limitations, including the high white matter retention and relatively low selectivity of tau over Aβ [152]. Then, two new derivatives: ^18^F-THK-5105 and ^18^F-THK-5117, were developed to improve the tau affinity and selectivity. These two compounds showed better imaging capability both in vitro and in vivo [45], but nonspecific binding in white matter still existed [46]. After that, ^18^F-THK-5351 was further developed. Encouragingly, ^18^F-THK-5351 showed favorable pharmacokinetics, higher affinity for tau protein fibrils, and lower retention in white matter, allowing for better tau visualization [47].

Using ^18^F-THK-5351, the spatial distribution of retention in AD patients is corresponded to the pattern reported by Braak previously [153]. In AD patients, significant higher retention is observed in association cortices and limbic areas than healthy subjects, and the level of tracer uptake was clearly associated with the cognitive function [154]. And by combining the topology information of tau pathology, various neurodegenerative diseases can be differentiated [155–157]. However, while ^18^F-THK-5351 seems to target similar binding site with ^18^F-AV-1451, it is less sensitive and specific to tau of AD and shows higher off-target binding than ^18^F-AV-1451 [48]. Additionally, the availability of monoamine oxidase B (MAO-B) was reported to affect the uptake of ^18^F-THK-5351 in brain [49]. So the retention in areas of commonly off-targeted and tissues containing MAO-B should be interpreted with caution.

### ^***11***^***C-PBB3: a potential tau-targeted PET tracer***

Another series of probes, phenyl/pyridinyl-butadienyl-benzothiazoles/benzothiazoliums (PBBs), were also developed as potential tau-targeted PET tracers. And among these derivatives, ^11^C-PBB3 was the most promising candidate, demonstrating a good binding affinity in the nanomolar range and excellent selectivity for tau over amyloid (40–50-fold) [50]. And compared to other classes of tracers, ^11^C-PBB3 is the first compound reported to detect a broad range of tau aggregates, binding other tau fibril types as well as AD PHFs [51]. Additionally, ^11^C-PBB3 has shown the potential to differentiate patients in the AD continuum group from biomarker-negative individuals and suspected non-AD pathophysiology group [158].

Nevertheless, some shortcomings of this tracer should be overcome in the future. While nonspecific signals of ^11^C-PBB3 are generally low, retention in venous sinuses is noticeable [50]. And ^11^C-PBB3 is vulnerable to light due to its structure [52]; the instability will add complexity to its synthesis, purification, and clinical usage. The short half-life of ^11^C also limits its availability. Besides, a radiometabolite of this tracer could enter the brain and confound the imaging results [159].

### *Limitations*

Now several kinds of ligands have been developed for tau imaging. But as tau proteins are heterogeneous in isoforms and conformations, and the roles of various tau pathologies are elusive, further researches are warranted to better classify these tracers and interpret the binding pattern of each tracer in longitudinal studies. And as quite a few studies have reported off-target retention of these tracers, more imaging-to-postmortem researches should be conducted to validate their binding characteristics. Since tau PET imaging is bound to be applied to monitor disease progression and therapeutic efficacy, reproducibility assessment of these compounds is critical for accurately comparing results. Besides, studies comparing the dynamic change of tau tracer retention and other biomarkers are necessary for a better exploration of the interactions of different pathology, as well as helping determining the best means used for disease evaluation at the right time.

## Glucose metabolism imaging

[^18^F]fuoro-2-deoxyglucose (^18^F-FDG) is another kind of tracer important in AD research. ^18^F-FDG is an analog of glucose, with the hydroxyl at C-2 position substituted by fluorine-18. ^18^F-FDG could reflect the glucose metabolism partly by responding to the hypoxia environment in some tumors, myocardial ischemia, and inflammation [160]. And as the 2-hydroxyl group is necessary for further glycolysis, ^18^F-FDG phosphorylated cannot be metabolized and is trapped in the cell until radioactive decay [161]. Hence, its retention is powerful to characterize tissues in terms of glucose metabolism. In addition to its extensive application in clinical cancer assessment, ^18^F-FDG also influences the field of neuroscience profoundly [162].

AD is a progressive neurodegenerative disease, so detecting the occurrence of neural dysfunction and assessing the severity of neurodegeneration are of great value both in researches and clinical practice. Using ^14^C-deoxyglucose, energy metabolism was reported to closely correlate to synaptic activation [163]. A literature of studies in recent years also suggest the glucose uptake characterized by ^18^F-FDG can be used to quantify brain connectivity [164]. Thus, hypometabolism is extensively utilized as a sensitive biomarker to evaluate brain function in AD and other dementias. In the new working criteria developed by NIA-AA, FDG was incorporated as a biomarker to assess the likelihood of AD pathology and stage of preclinical AD [13], while in the advancing diagnostic criteria of AD (IWG-2 criteria) proposed in 2014, hypometabolism was identified as a downstream topographical biomarker [9].

**Fig. 3:**
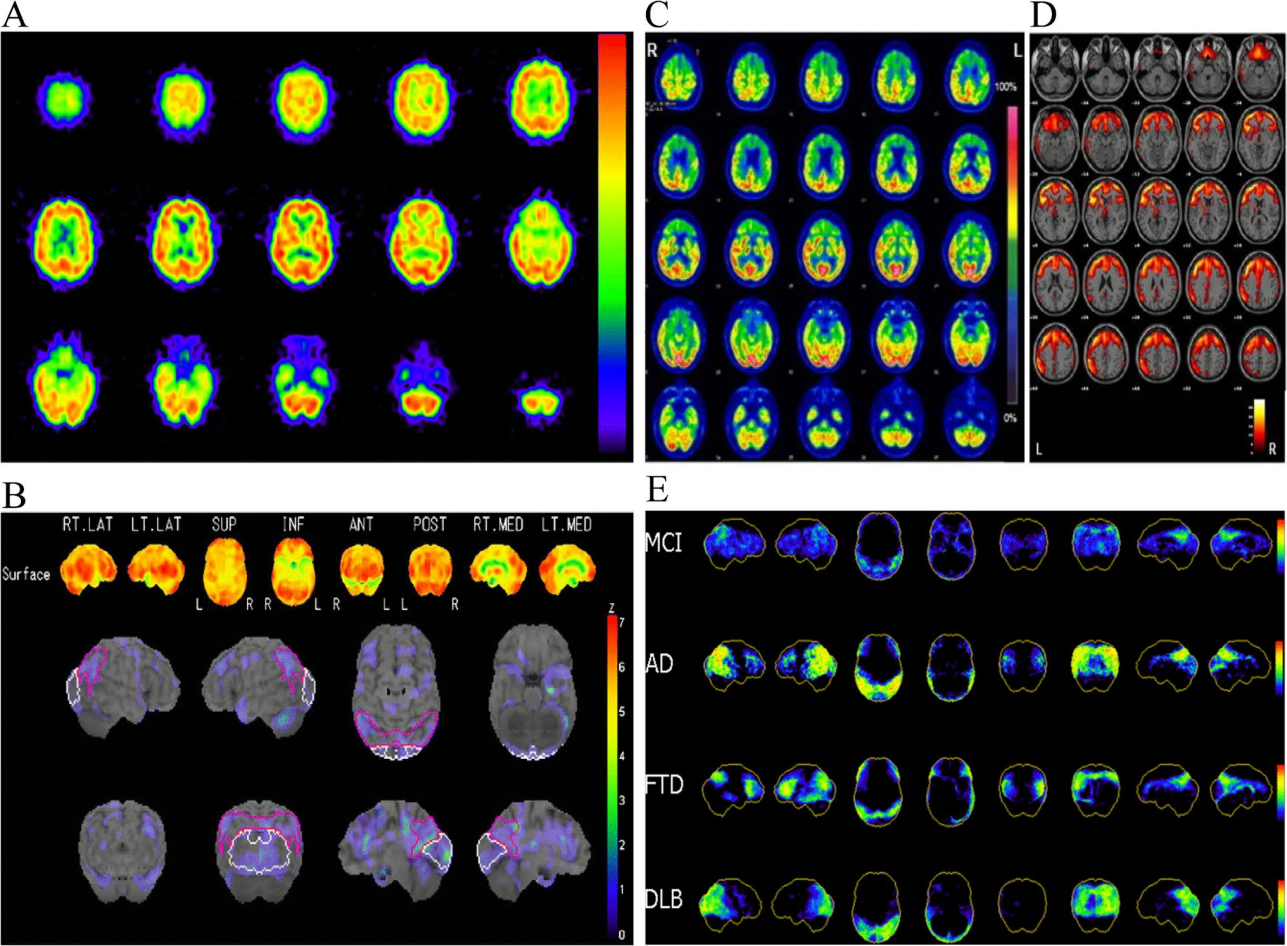
Examples of SPM and SSP. (**A**) Conventional IMP-SPECT image and (**B**) surface Z score map in a 73-year-old patient with AD. The slightly decreased perfusion in the brain regions of this AD patient was hard to detect by visual inspection, while surface Z score map automated showed brain regions with decreased perfusion and diagnosed this patient as AD. (RT. LAT right lateral, LT. LAT left lateral, SUP superior, INF inferior, ANT anterior, POST posterior, RT. MED right medial, LT. MED left medial, L left, R right); (**C**) Standard clinical display of FDG-PET images (“Standard FDG Images”) and (**D**) Voxel-based statistical parametric hypometabolic maps (“SPM Maps”) of a patient affected by behavioral variant of frontotemporal dementia. SPM Maps showed the abnormal brain regions more clearly. L = Left, R = right; (**E**) Representative cortical ^18^F-FDG PET patterns in NL, AD, DLB, and FTD. Three-dimensional stereotactic surface projections (3D-SSPs) and corresponding Z scores showing CMRglc reductions in clinical groups as compared with the NL database are displayed on a color-coded scale ranging from 0 (black) to 10 (red). From left to right: 3D-SSP maps are shown on the right and left lateral, superior and inferior, anterior and posterior, and right and left middle views of a standardized brain image (NL age-matched healthy elderly individuals, AD Alzheimer’s disease, DLB dementia with Lewy bodies, FTD frontotemporal dementia, CMRglc cerebral metabolic rate of glucose). (These researches were originally published in Eur J Nucl Med Mol Imaging. 2009 May;36(5):831–40., Neuroimage Clin. 2014;6:445–54. and J Nucl Med. 2008 Mar;49(3):390–8. Reprint permissions were obtained.)

However, hypometabolism in FDG PET is a sensitive but less specific biomarker, affected by age, depression, cognitive reserve, and many other factors [165]. Meanwhile, standardized FDG imaging acquisition protocol including scanning strategy and patient preparation also played an important role in image interpretation of AD patients [166]. Thus, the pattern of hypometabolism distribution across the whole brain should be taken into account when PET imaging results are interpreted. And considering the difficulty on discriminating delicate differences across various regions, using some automated quantitative techniques, like Statistical Parametric Mapping (SPM) and Stereotactic Surface Projections (SSP), may further provide complementary information and improve diagnostic accuracy (Fig. 3) [167–169]. It is reported that in AD patients, decreased glucose uptake in the parietotemporal areas and posterior cingulate cortices is a reliable hallmark of AD [170]. And there is evidence showing that areas presenting hypometabolism in AD patients are similar to those of default-model network [171]. The differences of hypometabolic pattern can also be used to differentiate AD from other neurodegenerative diseases [53–55].

In addition to the role of differentiating AD from healthy subjects and other dementias, another important application of ^18^F-FDG is to predict the conversion from MCI to AD. A recent study highlighted the ability of hypometabolism in middle and inferior temporal areas to predict the conversion from MCI to AD [56]. And according to a few studies, hypometabolism is the best predictor of AD converting among all biomarkers [54, 57]. All the evidence further suggests us to use FDG-PET measures to specifically rule out synaptic dysfunction and assess disease stages.

By using ^18^F-FDG, great progress in AD and other dementias has been made, but some insufficiency still exists. Firstly, cerebral FDG metabolism is such a sensitive biomarker that it is susceptible to many external factors. The variability in FDG PET imaging calls for more large-scaled and well-designed studies, to establish reasonable models and interpret PET results more accurately. As reported, SUV measurements might be more reproducible and reliable to interpret FDG PET images derived from PET/CT or PET/MRI, which could allow us to compare and evaluate patient’s PET results in a longitudinal time window or with other patient groups [172]. Although semiquantitative means used now greatly improve the diagnostic specificity, its sensitivity is less than visual evaluation by an expert [58]. Thus, visual means and semi-quantitative techniques should be weighed in future researches, and more advanced automated or semiautomated approaches should be developed. Besides, while ^18^F-FDG has become a powerful compound to measure synaptic dysfunction, it offers less information about disease etiology. To further promote the development of AD research, it seems more reasonable to combine FDG metabolism with other biomarkers and comprehensively understand the underlying pathophysiological mechanism of AD.

## Other targets for PET imaging

As the pathophysiology of AD is multifaceted and heterogeneous, the amyloid cascade hypothesis alone cannot fully elucidate it. There are also some other mechanisms proposed to play significant roles in the occurrence and progression of AD. Molecular imaging techniques, especially PET, are of great potential to investigate these pathophysiological processes and help to clarify their relationships with neuronal damage and cognitive dysfunction. We will discuss some other mechanisms involved in AD and list some radioligands targeting these alternations.

### *Neuroinflammation*

Neuroinflammation is regarded as an important participant in AD pathophysiology, though the exact role remains elusive. Some studies have suggested that the inflammation is involved in promoting tau hyperphosphorylation and driving neurodegenerative processes [173], while there is evidence showing its protective role at early AD stages [174]. Currently, numerous PET ligands are available to evaluate neuroinflammation by targeting underlying biological processes [175]. For example, microglial activation could be visualized by quantifying the 18-kDa translocator protein using radiotracers such as ^11^C-PK11195 [59], ^11^C-DAA1106 [60], ^18^F-FEDAA1106 [61], etc. ^11^C-DED can be utilized to bind monoamine oxidase B (MAO-B) and then measure reactive astrocytosis [62]. And the level of ^11^C-AA (arachidonic acid) uptake in brain is a reliable biomarker for detecting the up-regulation of phospholipase activity [63].

### *Metal ions in amyloid plaques*

High content of metal ions like Zn^2+^ and Cu^2+^ was observed in and around the formation site of amyloid plaques [176]. And these metals are able to mediate the protein aggregation and lead to the formation of amyloid plaques [177]. According to the data, a “Metal Hypothesis of AD” was proposed, and many ionophores and metal-chelators were developed as disease-modifying drugs [178]. The first PET radiotracer discovered to bind metal ions in amyloid plaques is ^18^F-CABS13, showing promising preliminary results in a mouse model [64]. And in recent years, ^11^C-L2-b and ^18^F-FL2-b were synthesized and evaluated to investigate the metallobiology of AD [65], but further researches on the metal-Aβ radioligands are warranted.

### *Glycogen synthase kinase 3*

Glycogen synthase kinase 3 (GSK3) is a wide expressed serine/threonine kinase involved in a range of cellular processes, including apoptosis, glucose regulation, and cell signaling [179–181]. It has been shown that GSK3 activity is associated with the formation of both amyloid plaques and NFTs [182, 183]. And inhibitors of GSK3 were reported to reduce Aβ production and tau aggregation [184]. These evidence encourage the “GSK3 hypothesis of AD” [185]. Some radiolabeled compounds are now available for GSK3 monitoring [186]. ^11^C-A1070722 and several ^11^C-labelling isonicotinamides were developed recently showing better binding characteristics than that synthesized previously [66, 67]. And more recently, glycogen synthase kinase 3β could be visualized by using ^18^F-labelling maleimide-based and isonicotinamide-based ligands which showed moderate brain uptake [68, 69].

### *Glutamatergic systems*

Glutamate is the most abundant excitatory neurotransmitter in brain, playing a vital role in learning and memory [187, 188]. Glutamate mediates ionotropic and metabotropic receptors (iGluRs and mGluRs, respectively), both of which are implicated in a spectrum of neurodegenerative diseases including AD [189, 190]. During the past decades, a number of radioligands have been developed for imaging iGluRs, but few PET tracers presented favorable in vivo results [70]. In terms of probes of mGluRs, no promising ligands are now available for imaging group III mGluRs (including mGluRs 4, 6, 7, and 8) and mGluR3. However, mGluR2 can be evaluated by a prospective tracer ^11^C-JNJ42491293. For visualizing mGluR1, ^18^F-FITM, ^11^C-ITMM, ^11^C-ITDM, and ^18^F-FIMX are clinically useful. And the most widely studied receptor, mGluR5, can be measured in vivo using ^18^F-FPEB, ^18^F-SP203, and ^11^C-ABP688 [71].

### *Endogenous cannabinoid system*

As a few alternations of the endogenous cannabinoid system (ECS) were reported in post-mortem studies [191], the ECS gets increasing attention as a novel target in some AD-therapeutic researches. Though the exact role of ECS in AD remains elusive, accumulating evidence suggests that the activation of cannabinoid signaling could alleviate AD symptoms by reducing excitotoxicity, neuroinflammation, and oxidative stress [192]. Currently, a series of compounds have been developed for the imaging of ECS. ^18^F-MK-9470 [72],^18^F-FMPEP-d2 [73], ^18^F-FPATPP [74], and ^11^C-SD5024 [75] are promising imaging tools for ECS researches in terms of type 1 cannabinoid receptor (CB1R). And the availability of type 2 cannabinoid receptor (CB2R) could be measured by several radioligands like ^18^F-labelling 1,3,5-triazine derivatives or ^11^C-labelling 4-oxoquinoline derivatives [76]. Fatty acid amide hydrolase (FAAH), the principal catabolic enzyme metabolizing endogenous cannabinoid, could also be detected using tracers like ^11^C-CURB [77], ^11^C-MK-3168 [78], and ^11^C-FAAH-1906 [79].

### *Cholinergic system*

The cholinergic dysfunction has also been found to be crucially involved in the pathophysiology of AD. A few post-mortem studies have suggested the cholinergic alternation in the course of dementia [193, 194]. And by using PET imaging techniques with selective tracers, diverse components of the cholinergic system could be measured in vivo [195]. Specifically, cerebral acetylcholinesterase (AChE) activity could be measured by using ^11^C-PMP [80] and ^11^C-MP4A [81]. ^11^C-MK-6884 [82] and ^18^F-ASEM [83] can be used to quantify the binding potential of the muscarinic and nicotinic acetylcholine receptors (mAChR and nAChR), respectively. And the imaging of vesicular acetylcholine transporter (VAChT) can be achieved by a series of compounds, such as ^11^C-TZ659 [84], ^18^F-VAT [85], and radiolabeled benzovesamicol VAChT analogs [86].

### *Monoaminergic system*

Although the cholinergic system is primarily affected in AD pathology, neurotransmitters of monoaminergic system are also implicated, including serotonin (5-HT), dopamine (DA), and norepinephrine (NE) [196, 197]. And the disruption of the dynamic balance of monoaminergic system may impair the functional neural networks of AD patients involved in affective regulation and executive function [198]. Serotonin 5-HT_1A_ receptor, which is particularly relevant to AD, could be selectively bound by ^11^C-WAY100635 [87] and ^18^F-MPPF [88]. And tracers targeting dopaminergic system have been extensively utilized in Parkinsonian disorders [199]. In terms of norepinephrine transporter imaging, ^11^C-MRB was considered as the most promising ligand [89].

## Conclusion

Multiple mechanisms are involved in the pathophysiological processes of AD, so a range of biomarkers are needed in the disease evaluation. The development of PET imaging technique has provided much crucial information about the occurrence and the progression of AD, contributing to the early and differential diagnosis of AD, as well as the assessment of therapeutic response. And the longitudinal scanning could help to clarify the dynamic change of each compound radiolabeled over the course of the disease. Besides, some biochemical processes unexplored could be visualized, which may contribute to the investigation of their role in AD etiology and the elucidation of the heterogeneity of clinical phenotypes. Therefore, PET imaging techniques are essential for the optimal AD evaluation and management in the future.

## References

[1] Khachaturian ZS (1985). Diagnosis of Alzheimer’s disease. Arch Neurol.

[2] Grasset L, Brayne C, Joly P, Jacqmin-Gadda H, Peres K, Foubert-Samier A (2016). Trends in dementia incidence: evolution over a 10-year period in France. Alzheimers Dement.

[3] Matthews FE, Stephan BC, Robinson L, Jagger C, Barnes LE, Arthur A (2016). A two decade dementia incidence comparison from the Cognitive Function and Ageing Studies I and II. Nat Commun.

[4] Satizabal CL, Beiser AS, Chouraki V, Chene G, Dufouil C, Seshadri S (2016). Incidence of dementia over three decades in the Framingham Heart Study. N Engl J Med.

[5] Wu YT, Fratiglioni L, Matthews FE, Lobo A, Breteler MM, Skoog I (2016). Dementia in western Europe: epidemiological evidence and implications for policy making. Lancet Neurol.

[6] Querfurth HW, Laferla FM (2010). Alzheimer’s disease. N Engl J Med.

[7] Villemagne VL (2016). Amyloid imaging: past, present and future perspectives. Ageing Res Rev.

[8] McKhann GM, Knopman DS, Chertkow H, Hyman BT, Jack CR, Kawas CH (2011). The diagnosis of dementia due to Alzheimer’s disease: recommendations from the National Institute on Aging-Alzheimer’s Association workgroups on diagnostic guidelines for Alzheimer’s disease. Alzheimers Dement.

[9] Dubois B, Feldman HH, Jacova C, Hampel H, Molinuevo JL, Blennow K (2014). Advancing research diagnostic criteria for Alzheimer’s disease: the IWG-2 criteria. Lancet Neurol.

[10] McKhann G, Drachman D, Folstein M, Katzman R, Price D, Stadlan EM (1984). Clinical diagnosis of Alzheimer’s disease: report of the NINCDS-ADRDA Work Group under the auspices of Department of Health and Human Services Task Force on Alzheimer’s Disease. Neurology.

[11] Villemagne VL, Rowe CC, Macfarlane S, Novakovic KE, Masters CL (2005). Imaginem oblivionis: the prospects of neuroimaging for early detection of Alzheimer’s disease. J Clin Neurosci.

[12] Tateno A, Sakayori T, Kawashima Y, Higuchi M, Suhara T, Mizumura S (2015). Comparison of imaging biomarkers for Alzheimer’s disease: amyloid imaging with [18F]florbetapir positron emission tomography and magnetic resonance imaging voxel-based analysis for entorhinal cortex atrophy. Int J Geriatr Psychiatry.

[13] Sperling RA, Aisen PS, Beckett LA, Bennett DA, Craft S, Fagan AM (2011). Toward defining the preclinical stages of Alzheimer’s disease: recommendations from the National Institute on Aging-Alzheimer’s Association workgroups on diagnostic guidelines for Alzheimer’s disease. Alzheimers Dement.

[14] Tian M, He X, Jin C, He X, Wu S, Zhou R (2021). Transpathology: molecular imaging-based pathology. Eur J Nucl Med Mol Imaging.

[15] Jacobs AH, Li H, Winkeler A, Hilker R, Knoess C, Ruger A (2003). PET-based molecular imaging in neuroscience. Eur J Nucl Med Mol Imaging.

[16] Phelps ME (2000). PET: the merging of biology and imaging into molecular imaging. J Nucl Med.

[17] Aschenbrenner AJ, Gordon BA, Benzinger TLS, Morris JC, Hassenstab JJ (2018). Influence of tau PET, amyloid PET, and hippocampal volume on cognition in Alzheimer disease. Neurology.

[18] de Wilde A, van der Flier WM, Pelkmans W, Bouwman F, Verwer J, Groot C (2018). Association of amyloid positron emission tomography with changes in diagnosis and patient treatment in an unselected memory clinic cohort: the ABIDE project. JAMA Neurol.

[19] Morris E, Chalkidou A, Hammers A, Peacock J, Summers J, Keevil S (2016). Diagnostic accuracy of (18)F amyloid PET tracers for the diagnosis of Alzheimer’s disease: a systematic review and meta-analysis. Eur J Nucl Med Mol Imaging.

[20] Albert MS, DeKosky ST, Dickson D, Dubois B, Feldman HH, Fox NC (2011). The diagnosis of mild cognitive impairment due to Alzheimer’s disease: recommendations from the National Institute on Aging-Alzheimer’s Association workgroups on diagnostic guidelines for Alzheimer’s disease. Alzheimers Dement.

[21] Benzinger TL, Blazey T, Jack CR, Koeppe RA, Su Y, Xiong C (2013). Regional variability of imaging biomarkers in autosomal dominant Alzheimer’s disease. Proc Natl Acad Sci U S A.

[22] Sperling RA, Karlawish J, Johnson KA (2013). Preclinical Alzheimer disease—the challenges ahead. Nat Rev Neurol.

[23] Fjell AM, Walhovd KB (2011). New tools for the study of Alzheimer’s disease: what are biomarkers and morphometric markers teaching us?. Neuroscientist.

[24] Klunk WE, Wang Y, Huang GF, Debnath ML, Holt DP, Mathis CA (2001). Uncharged thioflavin-T derivatives bind to amyloid-beta protein with high affinity and readily enter the brain. Life Sci.

[25] Hellwig S, Frings L, Bormann T, Vach W, Buchert R, Meyer PT (2019). Amyloid imaging for differential diagnosis of dementia: incremental value compared to clinical diagnosis and [(18)F]FDG PET. Eur J Nucl Med Mol Imaging.

[26] Zhang S, Smailagic N, Hyde C, Noel-Storr AH, Takwoingi Y, McShane R, et al. (11)C-PIB-PET for the early diagnosis of Alzheimer’s disease dementia and other dementias in people with mild cognitive impairment (MCI). Cochrane Database Syst Rev. 2014;2014:Cd010386. 10.1002/14651858.CD010386.pub2. 10.1002/14651858.CD010386.pub2 PMC6464750 25052054

[27] Choi SR, Golding G, Zhuang Z, Zhang W, Lim N, Hefti F (2009). Preclinical properties of 18F-AV-45: a PET agent for Abeta plaques in the brain. J Nucl Med: Off Publ Soc Nucl Med.

[28] Nelissen N, Van Laere K, Thurfjell L, Owenius R, Vandenbulcke M, Koole M (2009). Phase 1 study of the Pittsburgh compound B derivative 18F-flutemetamol in healthy volunteers and patients with probable Alzheimer disease. J Nucl Med: Off Publ Soc Nucl Med.

[29] Rowe CC, Ackerman U, Browne W, Mulligan R, Pike KL, O’Keefe G (2008). Imaging of amyloid beta in Alzheimer’s disease with 18F-BAY94-9172, a novel PET tracer: proof of mechanism. The Lancet Neurol.

[30] Mountz JM, Laymon CM, Cohen AD, Zhang Z, Price JC, Boudhar S (2015). Comparison of qualitative and quantitative imaging characteristics of [11C]PiB and [18F]flutemetamol in normal control and Alzheimer’s subjects. NeuroImage Clinical.

[31] Juréus A, Swahn BM, Sandell J, Jeppsson F, Johnson AE, Johnström P (2010). Characterization of AZD4694, a novel fluorinated Abeta plaque neuroimaging PET radioligand. J Neurochem.

[32] Cselényi Z, Jönhagen ME, Forsberg A, Halldin C, Julin P, Schou M (2012). Clinical validation of 18F-AZD4694, an amyloid-β-specific PET radioligand. J Nucl Med: Off Publ Soc Nucl Med.

[33] Rowe CC, Pejoska S, Mulligan RS, Jones G, Chan JG, Svensson S (2013). Head-to-head comparison of 11C-PiB and 18F-AZD4694 (NAV4694) for β-amyloid imaging in aging and dementia. J Nucl Med: Off Publ Soc Nucl Med.

[34] Magnusson K, Sehlin D, Syvänen S, Svedberg MM, Philipson O, Söderberg L (2013). Specific uptake of an amyloid-β protofibril-binding antibody-tracer in AβPP transgenic mouse brain. JAD.

[35] Fang XT, Hultqvist G, Meier SR, Antoni G, Sehlin D, Syvänen S (2019). High detection sensitivity with antibody-based PET radioligand for amyloid beta in brain. Neuroimage.

[36] Syvänen S, Hultqvist G, Gustavsson T, Gumucio A, Laudon H, Söderberg L (2018). Efficient clearance of Aβ protofibrils in AβPP-transgenic mice treated with a brain-penetrating bifunctional antibody. Alzheimer’s Res Therapy.

[37] Chien DT, Bahri S, Szardenings AK, Walsh JC, Mu F, Su MY (2013). Early clinical PET imaging results with the novel PHF-tau radioligand [F-18]-T807. JAD.

[38] Devous MD, Joshi AD, Navitsky M, Southekal S, Pontecorvo MJ, Shen H (2018). Test-retest reproducibility for the Tau PET imaging agent flortaucipir F 18. J Nucl Med: Off publ Soc Nucl Med.

[39] Southekal S, Devous MD, Kennedy I, Navitsky M, Lu M, Joshi AD (2018). Flortaucipir F 18 quantitation using parametric estimation of reference signal intensity. J Nucl Med: Off publ Soc Nucl Med.

[40] Ossenkoppele R, Rabinovici GD, Smith R, Cho H, Schöll M, Strandberg O (2018). Discriminative accuracy of [18F]flortaucipir Positron emission tomography for Alzheimer disease vs other neurodegenerative disorders. JAMA.

[41] Shcherbinin S, Schwarz AJ, Joshi A, Navitsky M, Flitter M, Shankle WR (2016). Kinetics of the Tau PET tracer 18F-AV-1451 (T807) in subjects with normal cognitive function, mild cognitive impairment, and Alzheimer disease. J Nucl Med: Off publ Soc Nucl Med.

[42] Lowe VJ, Curran G, Fang P, Liesinger AM, Josephs KA, Parisi JE (2016). An autoradiographic evaluation of AV-1451 Tau PET in dementia. Acta Neuropathol Commun.

[43] Marquié M, Verwer EE, Meltzer AC, Kim SJW, Agüero C, Gonzalez J (2017). Lessons learned about [F-18]-AV-1451 off-target binding from an autopsy-confirmed Parkinson’s case. Acta Neuropathol Commun.

[44] Chien DT, Szardenings AK, Bahri S, Walsh JC, Mu F, Xia C (2014). Early clinical PET imaging results with the novel PHF-tau radioligand [F18]-T808. JAD.

[45] Okamura N, Furumoto S, Harada R, Tago T, Yoshikawa T, Fodero-Tavoletti M (2013). Novel 18F-labeled arylquinoline derivatives for noninvasive imaging of tau pathology in Alzheimer disease. J Nucl Med: Off Publ Soc Nucl Med.

[46] Harada R, Okamura N, Furumoto S, Tago T, Yanai K, Arai H (2016). Characteristics of Tau and its ligands in PET imaging. Biomolecules.

[47] Harada R, Okamura N, Furumoto S, Furukawa K, Ishiki A, Tomita N (2016). 18F-THK5351: A novel PET radiotracer for imaging neurofibrillary pathology in Alzheimer disease. J Nucl Med: Off Publ Soc Nucl Med.

[48] Jang YK, Lyoo CH, Park S, Oh SJ, Cho H, Oh M (2018). Head to head comparison of [(18)F] AV-1451 and [(18)F] THK5351 for tau imaging in Alzheimer’s disease and frontotemporal dementia. Eur J Nucl Med Mol Imaging.

[49] Ng KP, Pascoal TA, Mathotaarachchi S, Therriault J, Kang MS, Shin M (2017). Monoamine oxidase B inhibitor, selegiline, reduces (18)F-THK5351 uptake in the human brain. Alzheimer’s Res Therapy.

[50] Maruyama M, Shimada H, Suhara T, Shinotoh H, Ji B, Maeda J (2013). Imaging of tau pathology in a tauopathy mouse model and in Alzheimer patients compared to normal controls. Neuron.

[51] Ono M, Sahara N, Kumata K, Ji B, Ni R, Koga S (2017). Distinct binding of PET ligands PBB3 and AV-1451 to tau fibril strains in neurodegenerative tauopathies. Brain : J Neurol.

[52] Hashimoto H, Kawamura K, Igarashi N, Takei M, Fujishiro T, Aihara Y (2014). Radiosynthesis, photoisomerization, biodistribution, and metabolite analysis of 11C-PBB3 as a clinically useful PET probe for imaging of tau pathology. J Nucl Med: Off Publ Soc Nucl Med.

[53] Mosconi L, Tsui WH, Herholz K, Pupi A, Drzezga A, Lucignani G (2008). Multicenter standardized 18F-FDG PET diagnosis of mild cognitive impairment, Alzheimer’s disease, and other dementias. J Nucl Med: Off Publ Soc Nucl Med.

[54] Perani D, Cerami C, Caminiti SP, Santangelo R, Coppi E, Ferrari L (2016). Cross-validation of biomarkers for the early differential diagnosis and prognosis of dementia in a clinical setting. Eur J Nucl Med Mol Imaging.

[55] Caminiti SP, Ballarini T, Sala A, Cerami C, Presotto L, Santangelo R (2018). FDG-PET and CSF biomarker accuracy in prediction of conversion to different dementias in a large multicentre MCI cohort. NeuroImage Clinical.

[56] Morbelli S, Bauckneht M, Arnaldi D, Picco A, Pardini M, Brugnolo A (2017). 18F-FDG PET diagnostic and prognostic patterns do not overlap in Alzheimer’s disease (AD) patients at the mild cognitive impairment (MCI) stage. Eur J Nucl Med Mol Imaging.

[57] Shaffer JL, Petrella JR, Sheldon FC, Choudhury KR, Calhoun VD, Coleman RE (2013). Predicting cognitive decline in subjects at risk for Alzheimer disease by using combined cerebrospinal fluid, MR imaging, and PET biomarkers. Radiology.

[58] Morbelli S, Brugnolo A, Bossert I, Buschiazzo A, Frisoni GB, Galluzzi S (2015). Visual versus semi-quantitative analysis of 18F-FDG-PET in amnestic MCI: an European Alzheimer’s Disease Consortium (EADC) project. JAD.

[59] Hashimoto K, Inoue O, Suzuki K, Yamasaki T, Kojima M (1989). Synthesis and evaluation of 11C-PK 11195 for in vivo study of peripheral-type benzodiazepine receptors using positron emission tomography. Ann Nucl Med.

[60] Yasuno F, Kosaka J, Ota M, Higuchi M, Ito H, Fujimura Y (2012). Increased binding of peripheral benzodiazepine receptor in mild cognitive impairment-dementia converters measured by positron emission tomography with [^11^C]DAA1106. Psychiatry Res.

[61] Varrone A, Mattsson P, Forsberg A, Takano A, Nag S, Gulyás B (2013). In vivo imaging of the 18-kDa translocator protein (TSPO) with [18F]FEDAA1106 and PET does not show increased binding in Alzheimer’s disease patients. Eur J Nucl Med Mol Imaging.

[62] Olsen M, Aguilar X, Sehlin D, Fang XT, Antoni G, Erlandsson A (2018). Astroglial responses to amyloid-beta progression in a mouse model of Alzheimer’s disease. Mol Imag Biol.

[63] Esposito G, Giovacchini G, Liow JS, Bhattacharjee AK, Greenstein D, Schapiro M (2008). Imaging neuroinflammation in Alzheimer’s disease with radiolabeled arachidonic acid and PET. J Nucl Med: Off Publ Soc Nucl Med.

[64] Vasdev N, Cao P, Van Oosten EM, Wilson AA, Houle S, Hao G (2012). Synthesis and PET imaging studies of [18F]2-fluoroquinolin-8-ol ([18F]CABS13) in transgenic mouse models of Alzheimer’s disease. MedChemComm.

[65] Cary BP, Brooks AF, Fawaz MV, Shao X, Desmond TJ, Carpenter GM (2015). Targeting metal-Aβ aggregates with bifunctional radioligand [(11)C]L2-b and a fluorine-18 analogue [(18)F]FL2-b. ACS Med Chem Lett.

[66] Gao M, Wang M, Zheng QH (2017). Synthesis of carbon-11-labeled isonicotinamides as new potential PET agents for imaging of GSK-3 enzyme in Alzheimer’s disease. Bioorg Med Chem Lett.

[67] Prabhakaran J, Zanderigo F, Sai KKS, Rubin-Falcone H, Jorgensen MJ, Kaplan JR (2017). Radiosynthesis and in vivo evaluation of [(11)C]A1070722, a high affinity GSK-3 PET tracer in primate brain. ACS Chem Neurosci.

[68] Hu K, Patnaik D, Collier TL, Lee KN, Gao H, Swoyer MR (2017). Development of [(18)F]maleimide-based glycogen synthase kinase-3β ligands for positron emission tomography imaging. ACS Med Chem Lett.

[69] Zhong Y, Yang S, Cui J, Wang J, Li L, Chen Y (2021). Novel (18)F-Labeled isonicotinamide-based radioligands for positron emission tomography imaging of glycogen synthase kinase-3β. Mol Pharm.

[70] Fu H, Chen Z, Josephson L, Li Z, Liang SH (2019). Positron emission tomography (PET) ligand development for ionotropic glutamate receptors: challenges and opportunities for radiotracer targeting N-methyl-d-aspartate (NMDA), α-amino-3-hydroxy-5-methyl-4-isoxazolepropionic acid (AMPA), and kainate receptors. J Med Chem.

[71] Fuchigami T, Nakayama M, Yoshida S (2015). Development of PET and SPECT probes for glutamate receptors. Sci World J.

[72] Burns HD, Van Laere K, Sanabria-Bohórquez S, Hamill TG, Bormans G, Eng WS (2007). [18F]MK-9470, a positron emission tomography (PET) tracer for in vivo human PET brain imaging of the cannabinoid-1 receptor. Proc Natl Acad Sci USA.

[73] Takkinen JS, López-Picón FR, Kirjavainen AK, Pihlaja R, Snellman A, Ishizu T (2018). [(18)F]FMPEP-d(2) PET imaging shows age- and genotype-dependent impairments in the availability of cannabinoid receptor 1 in a mouse model of Alzheimer’s disease. Neurobiol Aging.

[74] Lahdenpohja S, Rajala NA, Helin JS, Haaparanta-Solin M, Solin O, López-Picón FR (2020). Ruthenium-mediated (18)F-fluorination and preclinical evaluation of a new CB(1) receptor imaging agent [(18)F]FPATPP. ACS Chem Neurosci.

[75] Hjorth S, Karlsson C, Jucaite A, Varnäs K, Wählby Hamrén U, Johnström P (2016). A PET study comparing receptor occupancy by five selective cannabinoid 1 receptor antagonists in non-human primates. Neuropharmacology.

[76] Spinelli F, Capparelli E, Abate C, Colabufo NA, Contino M (2017). Perspectives of cannabinoid type 2 receptor (CB2R) ligands in neurodegenerative disorders: structure-affinity relationship (SAfiR) and structure-activity relationship (SAR) studies. J Med Chem.

[77] Wilson AA, Garcia A, Parkes J, Houle S, Tong J, Vasdev N (2011). [11C]CURB: Evaluation of a novel radiotracer for imaging fatty acid amide hydrolase by positron emission tomography. Nucl Med Biol.

[78] Liu P, Hamill TG, Chioda M, Chobanian H, Fung S, Guo Y (2013). Discovery of MK-3168: a PET tracer for imaging brain fatty acid amide hydrolase. ACS Med Chem Lett.

[79] Chen Z, Hou L, Gan J, Cai Q, Ye W, Chen J (2020). Synthesis and preliminary evaluation of a novel positron emission tomography (PET) ligand for imaging fatty acid amide hydrolase (FAAH). Bioorg Med Chem Lett.

[80] Kilbourn MR, Snyder SE, Sherman PS, Kuhl DE (1996). In vivo studies of acetylcholinesterase activity using a labeled substrate, N-[11C]methylpiperdin-4-yl propionate ([11C]PMP). Synapse (New York, NY).

[81] Shimada H, Hirano S, Sinotoh H, Ota T, Tanaka N, Sato K (2015). Dementia with Lewy bodies can be well-differentiated from Alzheimer’s disease by measurement of brain acetylcholinesterase activity-a [11C]MP4A PET study. Int J Geriatr Psychiatry.

[82] Li W, Wang Y, Lohith TG, Zeng Z, Tong L, Mazzola R, et al. The PET tracer [(11)C]MK-6884 quantifies M4 muscarinic receptor in rhesus monkeys and patients with Alzheimer’s disease. Sci Transl Med. 2022;14:eabg3684. 10.1126/scitranslmed.abg3684. 10.1126/scitranslmed.abg3684 35020407

[83] Coughlin JM, Rubin LH, Du Y, Rowe SP, Crawford JL, Rosenthal HB (2020). High availability of the α7-nicotinic acetylcholine receptor in brains of individuals with mild cognitive impairment: a pilot study using (18)F-ASEM PET. J Nucl Med: Off Publ, Soc Nucl Med.

[84] Jin H, Zhang X, Yue X, Liu H, Li J, Yang H (2016). Kinetics modeling and occupancy studies of a novel C-11 PET tracer for VAChT in nonhuman primates. Nucl Med Biol.

[85] Jin H, Yue X, Liu H, Han J, Flores H, Su Y (2018). Kinetic modeling of [(18) F]VAT, a novel radioligand for positron emission tomography imaging vesicular acetylcholine transporter in non-human primate brain. J Neurochem.

[86] Yue X, Jin H, Liu H, Luo Z, Zhang X, Kaneshige K (2017). Synthesis, resolution, and in vitro evaluation of three vesicular acetylcholine transporter ligands and evaluation of the lead fluorine-18 radioligand in a nonhuman primate. Org Biomol Chem.

[87] Raje S, Patat AA, Parks V, Schechter L, Plotka A, Paul J (2008). A positron emission tomography study to assess binding of lecozotan, a novel 5-hydroxytryptamine-1A silent antagonist, to brain 5-HT1A receptors in healthy young and elderly subjects, and in patients with Alzheimer's disease. Clin Pharmacol Ther.

[88] Vidal B, Sebti J, Verdurand M, Fieux S, Billard T, Streichenberger N (2016). Agonist and antagonist bind differently to 5-HT1A receptors during Alzheimer’s disease: a post-mortem study with PET radiopharmaceuticals. Neuropharmacology.

[89] Ding YS, Lin KS. PET imaging of the norepinephrine transporter: a review plus new developments. NeuroImage. 2006;31.

[90] Hamley IW. The amyloid beta peptide: a chemist’s perspective. Role in Alzheimer’s and fibrillization. Chem Rev. 2012;112:5147–92. 10.1021/cr3000994. 10.1021/cr3000994 22813427

[91] Priller C, Bauer T, Mitteregger G, Krebs B, Kretzschmar HA, Herms J (2006). Synapse formation and function is modulated by the amyloid precursor protein. J Neurosci.

[92] Turner PR, O’Connor K, Tate WP, Abraham WC (2003). Roles of amyloid precursor protein and its fragments in regulating neural activity, plasticity and memory. Prog Neurobiol.

[93] Blennow K, Mattsson N, Scholl M, Hansson O, Zetterberg H (2015). Amyloid biomarkers in Alzheimer’s disease. Trends Pharmacol Sci.

[94] Tarasoff-Conway JM, Carare RO, Osorio RS, Glodzik L, Butler T, Fieremans E (2015). Clearance systems in the brain—implications for Alzheimer disease. Nat Rev Neurol.

[95] Karran E, Mercken M, De Strooper B (2011). The amyloid cascade hypothesis for Alzheimer’s disease: an appraisal for the development of therapeutics. Nat Rev Drug Discov.

[96] Lee SJ, Nam E, Lee HJ, Savelieff MG, Lim MH (2017). Towards an understanding of amyloid-beta oligomers: characterization, toxicity mechanisms, and inhibitors. Chem Soc Rev.

[97] Jansen WJ, Ossenkoppele R, Tijms BM, Fagan AM, Hansson O, Klunk WE (2018). Association of cerebral amyloid-β aggregation with cognitive functioning in persons without dementia. JAMA Psychiat.

[98] Sarro L, Senjem ML, Lundt ES, Przybelski SA, Lesnick TG, Graff-Radford J (2016). Amyloid-β deposition and regional grey matter atrophy rates in dementia with Lewy bodies. Brain : J Neurol.

[99] Jagust W (2016). Is amyloid-beta harmful to the brain? Insights from human imaging studies. Brain.

[100] Jack CR, Knopman DS, Jagust WJ, Petersen RC, Weiner MW, Aisen PS (2013). Tracking pathophysiological processes in Alzheimer’s disease: an updated hypothetical model of dynamic biomarkers. Lancet Neurol.

[101] McGeer PL, McGeer EG (2013). The amyloid cascade-inflammatory hypothesis of Alzheimer disease: implications for therapy. Acta Neuropathol.

[102] Selkoe DJ (1991). The molecular pathology of Alzheimer’s disease. Neuron.

[103] Selkoe DJ (2002). Alzheimer’s disease is a synaptic failure. Science.

[104] Herrup K (2015). The case for rejecting the amyloid cascade hypothesis. Nat Neurosci.

[105] Rowe CC, Bourgeat P, Ellis KA, Brown B, Lim YY, Mulligan R (2013). Predicting Alzheimer disease with beta-amyloid imaging: results from the Australian imaging, biomarkers, and lifestyle study of ageing. Ann Neurol.

[106] Sperling RA, Johnson KA, Doraiswamy PM, Reiman EM, Fleisher AS, Sabbagh MN (2013). Amyloid deposition detected with florbetapir F 18 ((18)F-AV-45) is related to lower episodic memory performance in clinically normal older individuals. Neurobiol Aging.

[107] Villemagne VL, Pike KE, Chetelat G, Ellis KA, Mulligan RS, Bourgeat P (2011). Longitudinal assessment of Abeta and cognition in aging and Alzheimer disease. Ann Neurol.

[108] Okello A, Koivunen J, Edison P, Archer HA, Turkheimer FE, Nagren K (2009). Conversion of amyloid positive and negative MCI to AD over 3 years: an 11C-PIB PET study. Neurology.

[109] Strozyk D, Blennow K, White LR, Launer LJ (2003). CSF Abeta 42 levels correlate with amyloid-neuropathology in a population-based autopsy study. Neurology.

[110] Blennow K, Hampel H (2003). CSF markers for incipient Alzheimer’s disease. Lancet Neurol.

[111] Mattsson N, Insel PS, Landau S, Jagust W, Donohue M, Shaw LM (2014). Diagnostic accuracy of CSF Ab42 and florbetapir PET for Alzheimer’s disease. Ann Clin Transl Neurol.

[112] Maezawa I, Hong HS, Liu R, Wu CY, Cheng RH, Kung MP (2008). Congo red and thioflavin-T analogs detect Abeta oligomers. J Neurochem.

[113] Lockhart A, Lamb JR, Osredkar T, Sue LI, Joyce JN, Ye L (2007). PIB is a non-specific imaging marker of amyloid-beta (Abeta) peptide-related cerebral amyloidosis. Brain.

[114] Fodero-Tavoletti MT, Smith DP, McLean CA, Adlard PA, Barnham KJ, Foster LE (2007). In vitro characterization of Pittsburgh compound-B binding to Lewy bodies. J Neurosci.

[115] Ikonomovic MD, Klunk WE, Abrahamson EE, Mathis CA, Price JC, Tsopelas ND (2008). Post-mortem correlates of in vivo PiB-PET amyloid imaging in a typical case of Alzheimer’s disease. Brain.

[116] Vlassenko AG, McCue L, Jasielec MS, Su Y, Gordon BA, Xiong C (2016). Imaging and cerebrospinal fluid biomarkers in early preclinical Alzheimer disease. Ann Neurol.

[117] La Joie R, Ayakta N, Seeley WW, Borys E, Boxer AL, DeCarli C (2018). Multisite study of the relationships between antemortem [(11)C]PIB-PET Centiloid values and postmortem measures of Alzheimer’s disease neuropathology. Alzheimers Dement.

[118] Sperling RA, Laviolette PS, O’Keefe K, O’Brien J, Rentz DM, Pihlajamaki M (2009). Amyloid deposition is associated with impaired default network function in older persons without dementia. Neuron.

[119] Rowe CC, Ng S, Ackermann U, Gong SJ, Pike K, Savage G (2007). Imaging beta-amyloid burden in aging and dementia. Neurology.

[120] Hepp DH, Vergoossen DL, Huisman E, Lemstra AW, Netherlands Brain B, Berendse HW (2016). Distribution and load of amyloid-beta pathology in Parkinson disease and dementia with Lewy bodies. J Neuropathol Exp Neurol.

[121] Leuzy A, Carter SF, Chiotis K, Almkvist O, Wall A, Nordberg A (2015). Concordance and diagnostic accuracy of [11C]PIB PET and cerebrospinal fluid biomarkers in a sample of patients with mild cognitive impairment and Alzheimer’s disease. J Alzheimers Dis.

[122] Ostrom QT, Gittleman H, Liao P, Rouse C, Chen Y, Dowling J, et al. CBTRUS statistical report: primary brain and central nervous system tumors diagnosed in the United States in 2007–2011. Neuro-oncology. 2014;16 Suppl 4:iv1–63. 10.1093/neuonc/nou223. 10.1093/neuonc/nou223 PMC4193675 25304271

[123] Liu E, Schmidt ME, Margolin R, Sperling R, Koeppe R, Mason NS (2015). Amyloid-beta 11C-PiB-PET imaging results from 2 randomized bapineuzumab phase 3 AD trials. Neurology.

[124] Landau SM, Thomas BA, Thurfjell L, Schmidt M, Margolin R, Mintun M (2014). Amyloid PET imaging in Alzheimer’s disease: a comparison of three radiotracers. Eur J Nucl Med Mol Imaging.

[125] Dugger BN, Clark CM, Serrano G, Mariner M, Bedell BJ, Coleman RE (2014). Neuropathologic heterogeneity does not impair florbetapir-positron emission tomography postmortem correlates. J Neuropathol Exp Neurol.

[126] Seo SW, Ayakta N, Grinberg LT, Villeneuve S, Lehmann M, Reed B (2017). Regional correlations between [(11)C]PIB PET and post-mortem burden of amyloid-beta pathology in a diverse neuropathological cohort. Neuroimage Clin.

[127] Esparza TJ, Zhao H, Cirrito JR, Cairns NJ, Bateman RJ, Holtzman DM (2013). Amyloid-beta oligomerization in Alzheimer dementia versus high-pathology controls. Ann Neurol.

[128] Hatsuta H, Takao M, Ishii K, Ishiwata K, Saito Y, Kanemaru K (2015). Amyloid beta accumulation assessed with (1)(1)C-Pittsburgh compound B PET and postmortem neuropathology. Curr Alzheimer Res.

[129] Mathis CA, Kuller LH, Klunk WE, Snitz BE, Price JC, Weissfeld LA (2013). In vivo assessment of amyloid-beta deposition in nondemented very elderly subjects. Ann Neurol.

[130] Xu L, Wu X, Li R, Chen K, Long Z, Zhang J (2016). Prediction of progressive mild cognitive impairment by multi-modal neuroimaging biomarkers. J Alzheimers Dis.

[131] Iaccarino L, Chiotis K, Alongi P, Almkvist O, Wall A, Cerami C (2017). A cross-validation of FDG- and amyloid-PET biomarkers in mild cognitive impairment for the risk prediction to dementia due to Alzheimer’s disease in a clinical setting. J Alzheimers Dis.

[132] Nayate AP, Dubroff JG, Schmitt JE, Nasrallah I, Kishore R, Mankoff D (2015). Use of standardized uptake value ratios decreases interreader variability of [18F] florbetapir PET brain scan interpretation. AJNR Am J Neuroradiol.

[133] Wang Y, Mandelkow E (2016). Tau in physiology and pathology. Nat Rev Neurosci.

[134] Kevenaar JT, Hoogenraad CC (2015). The axonal cytoskeleton: from organization to function. Front Mol Neurosci.

[135] Ballatore C, Lee VM, Trojanowski JQ (2007). Tau-mediated neurodegeneration in Alzheimer’s disease and related disorders. Nat Rev Neurosci.

[136] Villemagne VL, Furumoto S, Fodero-Tavoletti M, Harada R, Mulligan RS, Kudo Y (2012). The challenges of Tau imaging. Future Neurol.

[137] Saroja SR, Sharma A, Hof PR, Pereira AC (2021). Differential expression of tau species and the association with cognitive decline and synaptic loss in Alzheimer’s disease. Alzheimer’s Dement: J Alzheimer’s Assoc.

[138] Ossenkoppele R, van der Kant R, Hansson O (2022). Tau biomarkers in Alzheimer’s disease: towards implementation in clinical practice and trials. Lancet Neurol.

[139] Karran E, Hardy J (2014). Antiamyloid therapy for Alzheimer’s disease—are we on the right road?. N Engl J Med.

[140] Blennow K, Hampel H, Weiner M, Zetterberg H (2010). Cerebrospinal fluid and plasma biomarkers in Alzheimer disease. Nat Rev Neurol.

[141] Fodero-Tavoletti MT, Furumoto S, Taylor L, McLean CA, Mulligan RS, Birchall I (2014). Assessing THK523 selectivity for tau deposits in Alzheimer’s disease and non-Alzheimer’s disease tauopathies. Alzheimer’s Res Therapy.

[142] Braak H, Braak E (1997). Frequency of stages of Alzheimer-related lesions in different age categories. Neurobiol Aging.

[143] Ben Bouallegue F, Mariano-Goulart D, Payoux P, Alzheimer’s disease neuroimaging I. Comparison of CSF markers and semi-quantitative amyloid PET in Alzheimer’s disease diagnosis and in cognitive impairment prognosis using the ADNI-2 database. Alzheimers Res Ther. 2017;9:32. 10.1186/s13195-017-0260-z. 10.1186/s13195-017-0260-z PMC5405503 28441967

[144] Jagust W (2014). Time for tau. Brain.

[145] Lohith TG, Bennacef I, Vandenberghe R, Vandenbulcke M, Salinas-Valenzuela C, Declercq R (2018). First-in-human brain imaging of Alzheimer dementia patients and elderly controls with (18)F-MK-6240, a PET tracer targeting neurofibrillary tangle pathology. J Nucl Med.

[146] Wong DF, Comley RA, Kuwabara H, Rosenberg PB, Resnick SM, Ostrowitzki S (2018). Characterization of 3 novel tau radiopharmaceuticals, (11)C-RO-963, (11)C-RO-643, and (18)F-RO-948, in healthy controls and in Alzheimer subjects. J Nucl Med.

[147] Chhatwal JP, Schultz AP, Marshall GA, Boot B, Gomez-Isla T, Dumurgier J (2016). Temporal T807 binding correlates with CSF tau and phospho-tau in normal elderly. Neurology.

[148] Johnson KA, Schultz A, Betensky RA, Becker JA, Sepulcre J, Rentz D (2016). Tau positron emission tomographic imaging in aging and early Alzheimer disease. Ann Neurol.

[149] Schwarz AJ, Yu P, Miller BB, Shcherbinin S, Dickson J, Navitsky M (2016). Regional profiles of the candidate tau PET ligand 18F-AV-1451 recapitulate key features of Braak histopathological stages. Brain.

[150] J C, K J. Human amyloid imaging conference 2015.www.alzforum.org/print-series/390816. 2015.

[151] Tian M, Civelek AC, Carrio I, Watanabe Y, Kang KW, Murakami K (2022). International consensus on the use of tau PET imaging agent (18)F-flortaucipir in Alzheimer’s disease. Eur J Nucl Med Mol Imaging.

[152] Dani M, Brooks DJ, Edison P (2016). Tau imaging in neurodegenerative diseases. Eur J Nucl Med Mol Imaging.

[153] Yokoi T, Watanabe H, Yamaguchi H, Bagarinao E, Masuda M, Imai K (2018). Involvement of the precuneus/posterior cingulate cortex is significant for the development of Alzheimer’s disease: a PET (THK5351, PiB) and resting fMRI study. Front Aging Neurosci.

[154] Kang JM, Lee SY, Seo S, Jeong HJ, Woo SH, Lee H (2017). Tau positron emission tomography using [(18)F]THK5351 and cerebral glucose hypometabolism in Alzheimer’s disease. Neurobiol Aging.

[155] Kikuchi A, Okamura N, Hasegawa T, Harada R, Watanuki S, Funaki Y (2016). In vivo visualization of tau deposits in corticobasal syndrome by 18F-THK5351 PET. Neurology.

[156] Ishiki A, Harada R, Okamura N, Tomita N, Rowe CC, Villemagne VL (2017). Tau imaging with [(18) F]THK-5351 in progressive supranuclear palsy. Eur J Neurol.

[157] Schaeverbeke J, Evenepoel C, Declercq L, Gabel S, Meersmans K, Bruffaerts R (2018). Distinct [(18)F]THK5351 binding patterns in primary progressive aphasia variants. Eur J Nucl Med Mol Imaging.

[158] Yousefzadeh-Nowshahr E, Winter G, Bohn P, Kneer K, von Arnim CAF, Otto M (2022). Quantitative analysis of regional distribution of tau pathology with 11C-PBB3-PET in a clinical setting. PLoS One.

[159] Kimura Y, Ichise M, Ito H, Shimada H, Ikoma Y, Seki C (2015). PET Quantification of Tau pathology in human brain with 11C-PBB3. J Nucl Med.

[160] Yao Y, Li Y-M, He Z-X, Civelek AC, Li X-F (2021). Likely common role of hypoxia in driving 18F-FDG uptake in cancer, myocardial ischemia, inflammation and infection. Cancer Biother Radiopharm.

[161] Ido TW, C-N.; Casella, V.; et. Labeled 2-deoxy-D-glucose analogs. 18F-Labeled 2-deoxy-2-fluoro-D-glucose, 2-deoxy-2-fluoro-D-mannose and 14C-2-deoxy-2-fluoro-D-glucose J Labelled Compd Radiopharm. 1978.

[162] Minoshima S, Cross D, Thientunyakit T, Foster NL, Drzezga A (2022). (18)F-FDG PET imaging in neurodegenerative dementing disorders: insights into subtype classification, emerging disease categories, and mixed dementia with copathologies. J Nucl Med: Off Publ Soc Nucl Med.

[163] Sokoloff L, Reivich M, Kennedy C, Des Rosiers MH, Patlak CS, Pettigrew KD (1977). The [14C]deoxyglucose method for the measurement of local cerebral glucose utilization: theory, procedure, and normal values in the conscious and anesthetized albino rat. J Neurochem.

[164] Yakushev I, Drzezga A, Habeck C (2017). Metabolic connectivity: methods and applications. Curr Opin Neurol.

[165] Kato T, Inui Y, Nakamura A, Ito K (2016). Brain fluorodeoxyglucose (FDG) PET in dementia. Ageing Res Rev.

[166] Jagust WJ, Bandy D, Chen K, Foster NL, Landau SM, Mathis CA (2010). The Alzheimer’s Disease Neuroimaging Initiative positron emission tomography core. Alzheimers Dement.

[167] Perani D, Della Rosa PA, Cerami C, Gallivanone F, Fallanca F, Vanoli EG (2014). Validation of an optimized SPM procedure for FDG-PET in dementia diagnosis in a clinical setting. Neuroimage Clin.

[168] Kim J, Cho SG, Song M, Kang SR, Kwon SY, Choi KH (2016). Usefulness of 3-dimensional stereotactic surface projection FDG PET images for the diagnosis of dementia. Medicine (Baltimore).

[169] Mosconi L, Tsui WH, Herholz K, Pupi A, Drzezga A, Lucignani G (2008). Multicenter standardized 18F-FDG PET diagnosis of mild cognitive impairment, Alzheimer’s disease, and other dementias. J Nucl Med.

[170] Mosconi L. Brain glucose metabolism in the early and specific diagnosis of Alzheimer’s disease. FDG-PET studies in MCI and AD. Eur J Nucl Med Mol Imaging. 2005;32:486–510. 10.1007/s00259-005-1762-7. 10.1007/s00259-005-1762-7 15747152

[171] Passow S, Specht K, Adamsen TC, Biermann M, Brekke N, Craven AR (2015). Default-mode network functional connectivity is closely related to metabolic activity. Hum Brain Mapp.

[172] Civelek A, Rana A, Malayeri A, Rodante J, Dey A, Jha A (2017). Intra and inter test reproducibility and comparison of PET-MRI and PET-CT derived 18F-FDG metric measurements. J Nucl Med.

[173] Maphis N, Xu G, Kokiko-Cochran ON, Cardona AE, Ransohoff RM, Lamb BT (2015). Loss of tau rescues inflammation-mediated neurodegeneration. Front Neurosci.

[174] Hamelin L, Lagarde J, Dorothee G, Leroy C, Labit M, Comley RA (2016). Early and protective microglial activation in Alzheimer’s disease: a prospective study using 18F-DPA-714 PET imaging. Brain.

[175] Zimmer ER, Leuzy A, Benedet AL, Breitner J, Gauthier S, Rosa-Neto P (2014). Tracking neuroinflammation in Alzheimer’s disease: the role of positron emission tomography imaging. J Neuroinflammation.

[176] Lovell MA, Robertson JD, Teesdale WJ, Campbell JL, Markesbery WR (1998). Copper, iron and zinc in Alzheimer’s disease senile plaques. J Neurol Sci.

[177] Ha C, Ryu J, Park CB (2007). Metal ions differentially influence the aggregation and deposition of Alzheimer’s beta-amyloid on a solid template. Biochemistry.

[178] Bush AI (2008). Drug development based on the metals hypothesis of Alzheimer’s disease. J Alzheimers Dis.

[179] Du J, Zhu X, Guo R, Xu Z, Cheng FF, Liu Q (2018). Autophagy induces G0/G1 arrest and apoptosis in menstrual blood-derived endometrial stem cells via GSK3-β/β-catenin pathway. Stem Cell Res Ther.

[180] Rao CV, Farooqui M, Madhavaram A, Zhang Y, Asch AS, Yamada HY. GSK3-ARC/Arg3.1 and GSK3-Wnt signaling axes trigger amyloid-β accumulation and neuroinflammation in middle-aged Shugoshin 1 mice. Aging Cell. 2020;19:e13221. 10.1111/acel.13221. 10.1111/acel.13221 PMC7576275 32857910

[181] Montori-Grau M, Tarrats N, Osorio-Conles O, Orozco A, Serrano-Marco L, Vázquez-Carrera M (2013). Glucose dependence of glycogen synthase activity regulation by GSK3 and MEK/ERK inhibitors and angiotensin-(1–7) action on these pathways in cultured human myotubes. Cell Signal.

[182] Phiel CJ, Wilson CA, Lee VM, Klein PS (2003). GSK-3alpha regulates production of Alzheimer’s disease amyloid-beta peptides. Nature.

[183] Hanger DP, Anderton BH, Noble W (2009). Tau phosphorylation: the therapeutic challenge for neurodegenerative disease. Trends Mol Med.

[184] Hurtado DE, Molina-Porcel L, Carroll JC, Macdonald C, Aboagye AK, Trojanowski JQ (2012). Selectively silencing GSK-3 isoforms reduces plaques and tangles in mouse models of Alzheimer’s disease. J Neurosci.

[185] Hooper C, Killick R, Lovestone S (2008). The GSK3 hypothesis of Alzheimer’s disease. J Neurochem.

[186] Pandey MK, DeGrado TR (2016). Glycogen synthase kinase-3 (GSK-3)-targeted therapy and imaging. Theranostics.

[187] Lovinger DM (2010). Neurotransmitter roles in synaptic modulation, plasticity and learning in the dorsal striatum. Neuropharmacology.

[188] Meldrum BS (2000). Glutamate as a neurotransmitter in the brain: review of physiology and pathology. J Nutr.

[189] Hamilton A, Zamponi GW, Ferguson SS (2015). Glutamate receptors function as scaffolds for the regulation of beta-amyloid and cellular prion protein signaling complexes. Mol Brain.

[190] Ribeiro FM, Vieira LB, Pires RG, Olmo RP, Ferguson SS (2017). Metabotropic glutamate receptors and neurodegenerative diseases. Pharmacol Res.

[191] Bedse G, Romano A, Lavecchia AM, Cassano T, Gaetani S (2015). The role of endocannabinoid signaling in the molecular mechanisms of neurodegeneration in Alzheimer’s disease. J Alzheimers Dis.

[192] Maroof N, Pardon MC, Kendall DA (2013). Endocannabinoid signalling in Alzheimer’s disease. Biochem Soc Trans.

[193] Davies P, Maloney AJ (1976). Selective loss of central cholinergic neurons in Alzheimer’s disease. Lancet.

[194] Davis KL, Mohs RC, Marin D, Purohit DP, Perl DP, Lantz M (1999). Cholinergic markers in elderly patients with early signs of Alzheimer disease. JAMA.

[195] Roy R, Niccolini F, Pagano G, Politis M (2016). Cholinergic imaging in dementia spectrum disorders. Eur J Nucl Med Mol Imaging.

[196] Lai MK, Tsang SW, Francis PT, Keene J, Hope T, Esiri MM (2002). Postmortem serotoninergic correlates of cognitive decline in Alzheimer’s disease. Neuro Report.

[197] Dringenberg HC (2000). Alzheimer’s disease: more than a ‘cholinergic disorder’—evidence that cholinergic-monoaminergic interactions contribute to EEG slowing and dementia. Behav Brain Res.

[198] Liu KY, Stringer AE, Reeves SJ, Howard RJ (2018). The neurochemistry of agitation in Alzheimer’s disease: a systematic review. Ageing Res Rev.

[199] Zhu L, Ploessl K, Kung HF (2014). PET/SPECT imaging agents for neurodegenerative diseases. Chem Soc Rev.

